# Label-Free Imaging of Single Nanoparticles Using Total Internal Reflection-Based Leakage Radiation Microscopy

**DOI:** 10.3390/nano10040615

**Published:** 2020-03-27

**Authors:** Liwen Jiang, Xuqing Sun, Hongyao Liu, Ruxue Wei, Xue Wang, Chang Wang, Xinchao Lu, Chengjun Huang

**Affiliations:** 1 Institute of Microelectronics of Chinese Academy of Sciences, Beijing 100029, China; jiangliwen@tju.edu.cn (L.J.); sunxuqing@ime.ac.cn (X.S.); liuhongyao@ime.ac.cn (H.L.); weiruxue@ime.ac.cn (R.W.); wangxue@ime.ac.cn (X.W.); wangchang@ime.ac.cn (C.W.); huangchengjun@ime.ac.cn (C.H.); 2 Currently with Center for Terahertz Waves, College of Precision Instrument and Optoelectronics Engineering, and the Key laboratory of Opto-electronics Information and Technology (Ministry of Education), Tianjin University, Tianjin 300072, China; 3 School of Microelectronics, University of Chinese Academy of Sciences, Beijing 100049, China

**Keywords:** total internal reflection, evanescent waves, leakage radiation, nanoparticles, label-free imaging, microscopy

## Abstract

Label-free, fast, and single nanoparticle detection is demanded for the in situ monitoring of nano-pollutants in the environment, which have potential toxic effects on human health. We present the label-free imaging of single nanoparticles by using total internal reflection (TIR)-based leakage radiation microscopy. We illustrate the imaging of both single polystyrene (PS) and Au nanospheres with diameters as low as 100 and 30 nm, respectively. As both far-field imaging and simulated near-field electric field intensity distribution at the interface showed the same characteristics, i.e., the localized enhancement and interference of TIR evanescent waves, we confirmed the leakage radiation, transforming the near-field distribution to far-field for fast imaging. The localized enhancement of single PS and Au nanospheres were compared. We also illustrate the TIR-based leakage radiation imaging of single polystyrene nanospheres with different incident polarizations. The TIR-based leakage radiation microscopy method is a competitive alternative for the fast, in situ, label-free imaging of nano-pollutants.

## 1. Introduction

Due to their unique properties among bulk materials, synthetic nanoparticles play a key role in various applications, such as disease diagnosis [1,2], cancer treatment [3,4], and drug delivery [5], leading to the growing amount of nanoparticles being released during industrial processes. On the other hand, nanoparticles occurring naturally, such as viruses, may introduce severe diseases and environmental deterioration. In consequence, there have been increasing concerns over the potential toxic effects of nano-pollutants on human health and the environment [6,7,8]. Due to the demand for recognizing nano-pollutants in the environment, a label-free, fast, in situ, single nanoparticle detection method is highly desired.

Benefitting from the high sensitivity induced by the interaction between single nanoparticles and evanescent waves, numerous studies on label-free single nanoparticle imaging have been explored. Total internal reflection (TIR)-based dark-field microscopy utilizes TIR illumination to excite evanescent waves and collect the particle scattering initiated by the evanescent waves for imaging. He et al. achieved the label-free tracking of single 40 nm Au particles in solution and a live HeLa cell membrane [9]. In addition, Enoki et al. achieved the label-free imaging of the single influenza virus [10]. Compared with traditional microscopy, TIR-based dark-field microscopy needs a device to separate the scattering from the reflection background for increasing the signal-to-noise ratio, which increases the complexity of the experimental system. Leakage radiation microscopy is also a label-free imaging method that utilizes the inherent leakage radiation of surface plasmon polaritons (SPPs), directly imaging near-field SPPs propagating at the air–metal interface to far-field [11]. By imaging the interaction between SPPs and adsorbed objects at the interface, leakage radiation microscopy is used for the fast and label-free detection of single polystyrene (PS) and silica nanoparticles [12,13,14], the single influenza virus [15], single exosomes [16], and single DNA molecules [17], as well as tracking the membrane proteins [18] and organelles [19] in living cells. Although leakage radiation microscopy is label-free and high sensitivity, the Au film for SPP excitation imposes a strict limitation on the substrate. Additionally, only the transverse magnetic (TM)-polarized illumination for SPP excitation can activate the leakage radiation microscopy. 

We know that the leakage radiation of SPPs transforms the free electron density fluctuation on metal surfaces to far-field. In this work, we found the leakage radiation of TIR evanescent waves on glass surfaces even if no such free electrons existed in the glass substrate. We have introduced TIR-based leakage radiation microscopy for the fast and label-free imaging of single nanoparticles, and present the imaging of single PS and Au nanospheres with diameters as low as 100 and 30 nm, respectively. By analyzing the similar characteristics of experimental far-field imaging and simulated near-field electric field intensity distribution, the leakage radiation of TIR evanescent waves was confirmed. TIR-based leakage radiation microscopy has no requirement for the scattering separation device and the metallic substrate, and can achieve the imaging by both TM and transverse electric (TE)-polarized illumination, which makes it more versatile, much simpler, and lower cost compared to TIR dark-field microscopy and leakage radiation microscopy. This method is a potential candidate for applications in the fast, in situ, label-free imaging of nano-pollutants. 

## 2. Materials and Methods 

**Figure 1:**
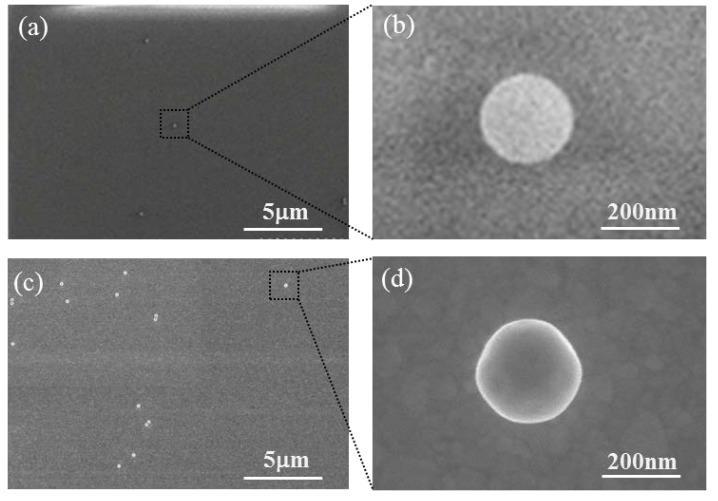
SEM imaging of (**a**) dispersed 200 nm PS nanospheres, (**b**) a single 200 nm PS nanosphere, (**c**) dispersed 200 nm Au nanospheres, and (**d**) a single 200 nm Au nanosphere.

For the single nanoparticle imaging, we deposited PS and Au nanospheres dispersedly on cover glass surfaces. The concentration of the purchased PS nanosphere solutions (Alfa Aesar Ltd., Beijing, China) was 2.5 wt.%, and they were diluted with ethanol to 1.79 × 10^−3^ mg/mL. The optical density of the Au nanosphere solutions (Nanoseedz Ltd., Hongkong, China) was 5 cm^−1^, and they were diluted with ethanol to 2.90 × 10^−3^ mg/mL, 2.10 × 10^−4^ mg/mL, 9.00 × 10^−5^ mg/mL, and 4.00 × 10^−5^ mg/mL for the diameters of 200, 100, 60, and 30 nm, respectively. During the deposition, we put a sapphire cover glass (*n* = 1.78) on a hotplate and preheated it to 423 K for 5 min. After this, we dripped one drop of the diluted nanosphere solution onto the cover glass. The nanospheres were situated dispersedly with the evaporation of the ethanol. Figure 1 shows the scanning electron microscope (SEM) imaging of the dispersed 200 nm PS and 200 nm Au nanospheres on the cover glass.

**Figure 2:**
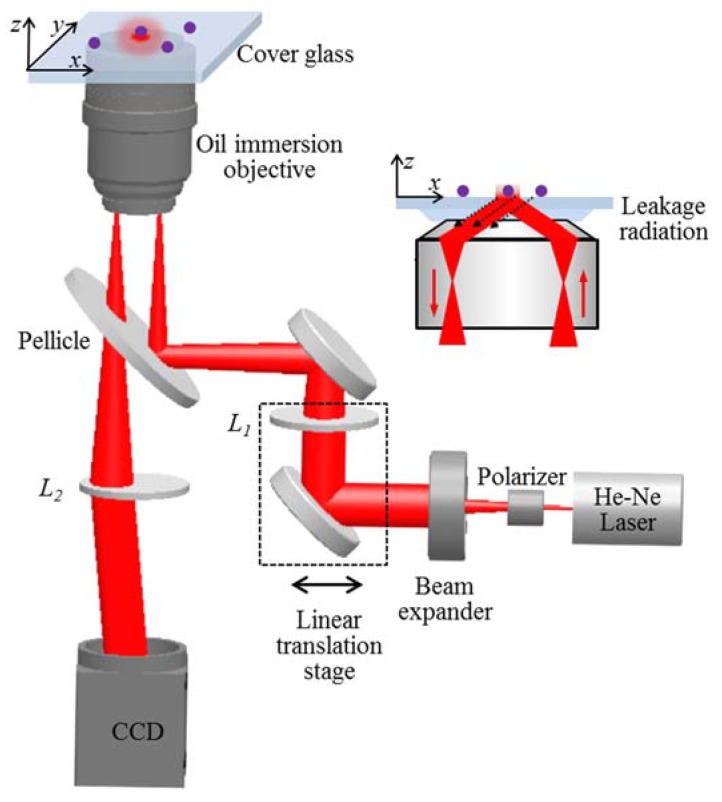
The experimental setup for the total internal reflection (TIR)-based leakage radiation microscopy. The inset shows the mechanism of the TIR-based leakage radiation.

The experimental setup for the TIR-based leakage radiation microscopy is shown in Figure 2, in which an objective-based TIR illumination system was built. A He–Ne laser with a 633 nm wavelength was used for the light source. The laser beam was collimated and expanded by a beam expander. A polarizer was inserted to deliver TM or TE illumination. The incident angle for the TIR illumination was controlled by lens *L*_1_, mounted on a linear translation stage that changed the displacement of the incident light to the objective axis. The evanescent waves were excited at the glass–air interface and interacted with the single nanospheres. Then, the scattered evanescent waves were collected with reflection by the objective, as shown in the inset of Figure 2. Finally, the collected light was transmitted through the pellicle and tube lens *L*_2_ and was imaged by a charge-coupled device (CCD) camera to obtain far-field imaging of the single nanospheres. A post-processing was required to increase the imaging contrast by eliminating the reflection background. Two cycles with an average of twenty measurements were acquired; one was the sample imaging with nanospheres, the other was the reference imaging without nanospheres. After subtracting the reference imaging and the CCD dark noise from the sample imaging with Image J software (1.52h), we obtained the imaging of single nanospheres.

## 3. Results and Discussion

**Figure 3:**
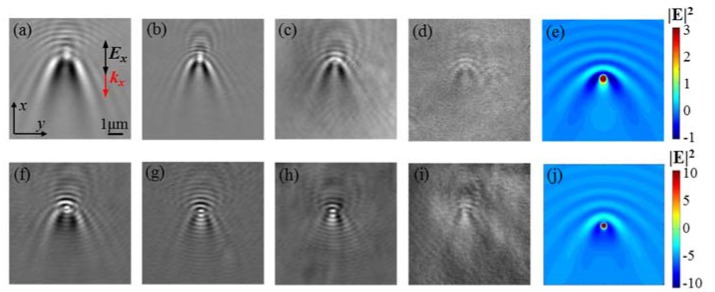
The TIR-based leakage radiation imaging of single polystyrene (PS) nanospheres under TM illumination with diameters of (**a**) 500, (**b**) 400, (**c**) 200, and (**d**) 100 nm. The imaging of single Au nanospheres under TM illumination with diameters of (**f**) 200, (**g**) 100, (**h**) 60, and (**i**) 30 nm. The simulated near-field electric field intensity of a single (**e**) 200 nm PS nanosphere and (**j**) 200 nm Au nanosphere. The *k_x_* indicates the propagation direction of the evanescent waves.

Figure 3a–d shows the imaging of single PS nanospheres with diameters of 500, 400, 200, and 100 nm under TM illumination. All the images show similar characteristics to those of SPP-based leakage radiation imaging [13,14,15,16]. The central bright dot and the parabolic fringes were introduced by the localized enhancement and interference of evanescent waves. During the propagation on the interface, the evanescent waves interacted with the single nanospheres and induced localized enhancement within the gap between the nanospheres and the substrate. Meanwhile, the nanospheres reradiated the in-plane scattered evanescent waves that interfered with the incidence and generated tails along the propagation direction as well as interference fringes in the opposite direction. Finally, the localized enhancement and interference fringes at the interface were transformed to far-field by leakage radiation for fast imaging. In Figure 3f–i, we also obtained TM-illuminated TIR-based leakage radiation imaging of single Au nanospheres with diameters of 200, 100, 60, and 30 nm. Similar to the PS nanospheres, all of the images show the bright dot and parabolic fringes. The tail intensity of the PS nanospheres along the evanescent wave propagation direction was stronger than that of the Au nanospheres, because more evanescent waves propagated through the PS nanosphere with less attenuation.

We simulated the electric field intensity |*E*|^2^ near the interface by using commercial finite difference time domain (FDTD) software (Vancouver, Canada, v2018a (8.19.1584)) from Lumerical Solutions, Inc. [20]. The total field scattered field (TFSF) plane wave was used as the simulation source. The simulation mesh was 5 nm × 5 nm × 5 nm, and the glass–air interface was set as a *z* = 0 nm plane. The single nanoparticle was positioned at the center of the interface (0, 0, 0). The near-field monitor was set at *z* = 5 nm apart from the interface. The index parameters were set as *n*_air_ = 1 and *n*_glass_ = 1.78. The perfectly matched layer (PML) was set as the simulation boundary condition. Similar to the experiment, we conducted two simulations: a sample with nanospheres and a reference without them. After subtracting the reference simulation from the sample, the near-field |*E*|^2^ of single PS and Au nanospheres with diameters of 200 nm at *z* = 5 nm is shown in Figure 3e,j. The bright dots with tails and parabolic interference fringes shown in the near-field |*E*|^2^ are the same features shown in the far-field imaging. Therefore, the leakage radiation of TIR evanescent waves projecting the near-field evanescent waves to far-field is confirmed. 

**Figure 4:**
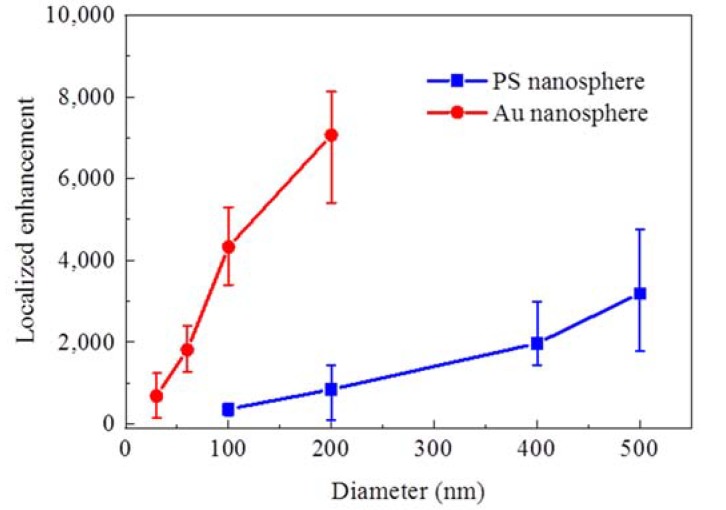
The diameter-dependent localized enhancement of single PS and Au nanospheres under TM illumination.

To quantitatively analyze the interaction between TIR evanescent waves and single nanoparticles, we found the localized enhancement by extracting the area of the bright dot in the images and adding grey values within the area. In Figure 4, the localized enhancement of PS and Au nanospheres of different diameters under TM illumination is compared, and both the PS and Au nanospheres show increasing enhancement at larger sizes. The Au nanospheres possessed a higher localized enhancement compared to the PS nanospheres of the same diameter, which was mainly due to the stronger polarization of the Au nanoparticles interacting with the evanescent waves.

**Figure 5:**
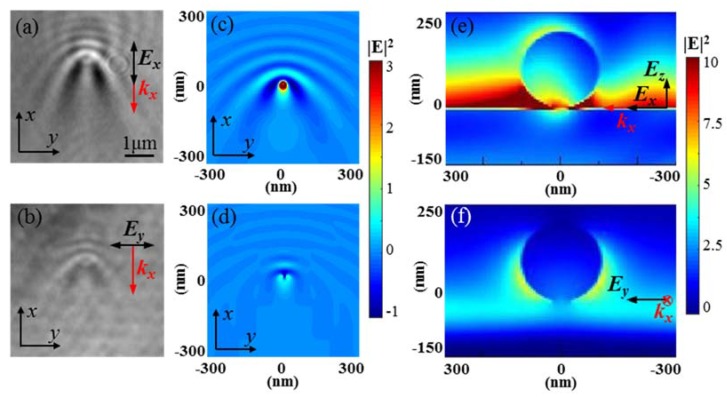
The TIR-based leakage radiation imaging of single 200 nm PS nanospheres under (**a**) TM illumination and (**b**) TE illumination. The simulated near-field electric field intensity of 200 nm PS nanospheres at *z* = 5 nm under (**c**) TM illumination and (**d**) TE illumination. The simulated near-field electric field intensity of 200 nm PS nanospheres at (**e**) *y* = 0 nm under TM illumination, and (**f**) *x* = 0 nm under TE illumination. The *k_x_* indicates the propagation direction of the evanescent waves.

In Figure 5, we compare the TIR-based leakage radiation imaging to single 200 nm PS nanospheres under TM and TE illumination. In Figure 5a,b, the imaging with TM illumination has higher sensitivity with brighter dots and more obvious interference fringes compared with those under TE illumination. In Figure 5c,d, we simulate the near-field electric field intensity |*E*|^2^ at *z* = 5 nm under both TM and TE illumination, which shows consistency with the experiment results. In Figure 5e,f, we also simulate |*E*|^2^ at the plane of *y* = 0 nm under TM illumination and *x* = 0 nm under TE illumination, with the plane parallel to the electric field. The evanescent electric field excited by the TM illumination had both *x* and *z* components *E_x_* and *E_z_*, while the TE illumination excited only the *y* component *E_z_* [21]. The TM illumination excited stronger evanescent waves at the interface and induced localized enhancement around the gap between the nanospheres and the substrate. Yet, the weak TE-polarized evanescent waves induced the nanospheres to radiate as a dipole and led to weak localized enhancement. We also found more interference fringes under TM illumination compared those under TE illumination, which resulted from the longer Goos–Hanchen shift under TM illumination [22,23].

## 4. Conclusions

In summary, we presented an alternative approach for the fast, in situ, and label-free imaging of single nanoparticles using TIR-based leakage radiation microscopy. Far-field imaging of single PS and Au nanospheres was obtained. As both the far-field imaging and simulated near-field electric field intensity showed the same features, we found the leakage radiation of TIR evanescent waves that projects the near-field distribution to far-field imaging. We also found stronger localized enhancement of Au nanospheres compared with that of PS nanospheres. The TIR-based leakage radiation imaging with TM and TE illumination were compared, and the higher sensitivity with TM illumination imaging was illustrated. TIR-based leakage radiation microscopy is a potential candidate for applications in the fast, in situ, label-free imaging of nano-pollutants, which it is helpful to the environment to control and monitor.
